# A Presenilin/Notch1 pathway regulated by *miR-375*, *miR-30a*, and *miR-34a* mediates glucotoxicity induced-pancreatic beta cell apoptosis

**DOI:** 10.1038/srep36136

**Published:** 2016-11-02

**Authors:** Yating Li, Tao Zhang, Yuncai Zhou, Yi Sun, Yue Cao, Xiaoai Chang, Yunxia Zhu, Xiao Han

**Affiliations:** 1 Key Laboratory of Human Functional Genomics of Jiangsu Province, Department of Biochemistry and Molecular Biology, Nanjing Medical University, Nanjing, Jiangsu, China

## Abstract

The presenilin-mediated Notch1 cleavage pathway plays a critical role in controlling pancreatic beta cell fate and survival. The aim of the present study was to investigate the role of Notch1 activation in glucotoxicity-induced beta cell impairment and the contributions of *miR-375*, *miR-30a*, and *miR-34a* to this pathway. We found that the protein levels of presenilins (PSEN1 and PSEN2), and NOTCH1 were decreased in INS-1 cells after treatment with increased concentrations of glucose, whereas no significant alteration of mRNA level of *Notch1* was observed. Targeting of *miR-375*, *miR-30a*, and *miR-34a* to the 3′utr of *Psen1*, *Psen2*, and *Notch1*, respectively, reduced the amounts of relevant proteins, thereby reducing NICD1 amounts and causing beta cell apoptosis. Overexpression of NICD1 blocked the effects of glucotoxicity as well as miRNA overabundance. Downregulating the expression of *miR-375*, *miR-30a*, and *miR-34a* restored PSEN1, PSEN2, and NICD1 production and prevented glucotoxicity-induced impairment of the beta cells. These patterns of miRNA regulation of the Notch1 cleavage pathway were reproduced in GK rats as well as in aged rats. Our findings demonstrated that miRNA-mediated suppression of NICD1 links the presenilin/Notch1 pathway to glucotoxicity in mature pancreatic beta cells.

Decreases in pancreatic beta cell function and mass are hallmarks of the progression of type 2 diabetes^1^,^2^, with glucotoxicity serving as a critical contributor to beta cell impairment observed in diabetic patients as well as in rodents^3^,^4^. Prolonged exposure of beta cells to elevated concentrations of glucose leads to decreases in glucose-stimulated insulin secretion (GSIS), inhibition of insulin gene expression, and induction of beta cell death by apoptosis. These effects of glucotoxicity are thought to involve several mechanisms, including oxidative stress, endoplasmic reticulum (ER) stress, and inflammation^5^,^6^,^7^. Numerous functional genes related to glucotoxicity-induced beta cell failure have been identified, including *Neurod1*, *Pdx1*, and *Bcl2*, but additional genes still require investigation. One of these is the *Notch1* gene.

Notch1 is a transmembrane receptor that plays a critical role in controlling cell fate during developmental processes, including those occurring in pancreatic tissues^8^. Notch1 is activated by interacting with its ligands (Delta or Jagged) located on adjacent cell surfaces and then undergoes intercellular proteolytic cleavage to generate the Notch1 intercellular domain (NICD1), which regulates cell differentiation, proliferation, and apoptosis. The complete cleavage and activation of Notch1 is mediated by the γ-secretase enzyme complex, consisting of presenilins, nicastrin (NCSTN), presenilin enhancer 2 (PEN2), and anterior pharynx-defective 1 (APH1)^9^,^10^.

The presenilins are critical catalytic subunits of γ-secretase and are implicated in vesicular trafficking, calcium homeostasis, and the regulation of apoptosis^11^,^12^. Their functional role is not well defined, but their presence of both presenilins and γ-secretase has been reported in beta cells^12^,^13^. Current evidence indicates that PSEN1 can promote beta cell survival via the cleavage of Notch1 in both adult human and mouse pancreatic islet cells^14^. Moreover, members of the Notch pathway are upregulated by the cytokine IL-1β in both rat primary islets and INS-1E cells^15^. The existing evidence therefore supports the retention of an intact presenilin/Notch1 pathway in mature pancreatic beta cells. However, the mechanisms that regulate the presenilin/Notch1 pathway in beta cells remain poorly understood.

One potential regulatory mechanism is via microRNAs (miRNAs). These are endogenous noncoding RNAs (~22 nucleotides) that regulate gene expression by binding to the 3′utr of their target mRNAs, resulting in degradation and/or translational inhibition of potentially hundreds of target mRNAs^16^. RNA sequencing and expression studies have identified several miRNAs expressed in pancreatic islets, including *miR-375*, *miR-30a*, and *miR-34a*^16^,^17^. Genetic studies have shown that *miR-375* knockout mice develop hyperglycemia and show reduced beta cell mass^18^, whereas *miR-375* overabundance suppresses GSIS and beta cell survival^19^,^20^. *MiR-30a* has been associated with glucotoxicity-induced defects in insulin secretion^17^. A role for *miR-34a* has also been repeatedly reported in both type 1 and type 2 diabetes as well as in age-associated diabetes^21^,^22^,^23^. The effects of *miR-375*, *miR-30a*, and *miR-34a* have been examined in pancreatic islet cells under diabetic conditions, but their contributions to a specific pathway have never been reported. The present study investigated the potential role of *miR-375, miR-30a*, and *miR-34a* on regulation of the presenilin/NOTCH1 pathway in mature pancreatic beta cells.

## Results

### Decreased γ-secretase-mediated Notch1 cleavage by glucotoxicity

**Figure 1:**
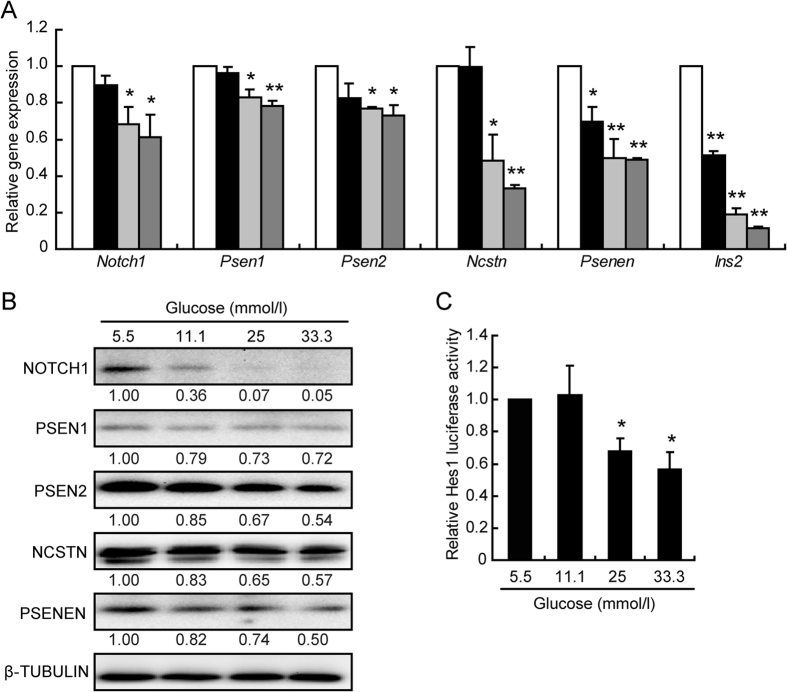
Notch1 cleavage was decreased by glucotoxicity. The mRNA (**A**) and protein (**B**) levels were analyzed in INS-1 cells treated for 24 h with different glucose concentrations (mmol/l): 5.5 (control, white bar), 11.1 (black bar), 25 (light grey bar), and 33.3 (heavy grey bar). Quantitative measurements were obtained by gray intensity analysis relative to β-TUBULIN. Relative luciferase activity of the Hes-1 promoter (**C**) in INS-1 cells treated with 5.5 mmol/l glucose (control) or the indicated glucose concentration. Data shown are means ± SEM and are representative of three separate experiments. *p < 0.05 and **p < 0.01 *vs*. control.

An involvement of notch1 signaling in glucotoxicity-induced beta cell impairment was explored by determining the mRNA levels and protein levels of *Notch1* and the main components of γ-secretase (*Psen1*, *Psen2*, *Ncstn*, and *Psenen*) (Fig. 1A,B). The mRNA levels of *Notch1*, *Psen1*, and *Psen2* were not altered following the 11.1 mmol/l glucose treatment and were only slightly decreased after 24 h exposure to 25 and 33.3 mmol/l glucose. The gene expression levels of *Ncstn*, *Psenen*, and *Ins2* were significantly decreased in a dose-dependent manner, (except for the alteration in *Ncstn* expression observed with 11.1 mmol/l glucose) (Fig. 1A). The protein levels were significantly decreased in a dose-dependent manner, with a particularly dramatic decrease observed in the level of NICD1, the active form of Notch1 (Fig. 1B).

The effect of high glucose on Notch1 signaling was further examined using a luciferase reporter carrying the active site of Hes1, a major downstream regulator of Notch1. The Hes1 activity was decreased after a 12 h treatment with glucose concentrations greater than 25 mmol/l (Fig. 1C). All the data suggest that Notch1 signaling can be repressed by glucotoxicity.

### Decreased Notch1 cleavage mediated glucotoxicity-induced beta cell apoptosis

**Figure 2:**
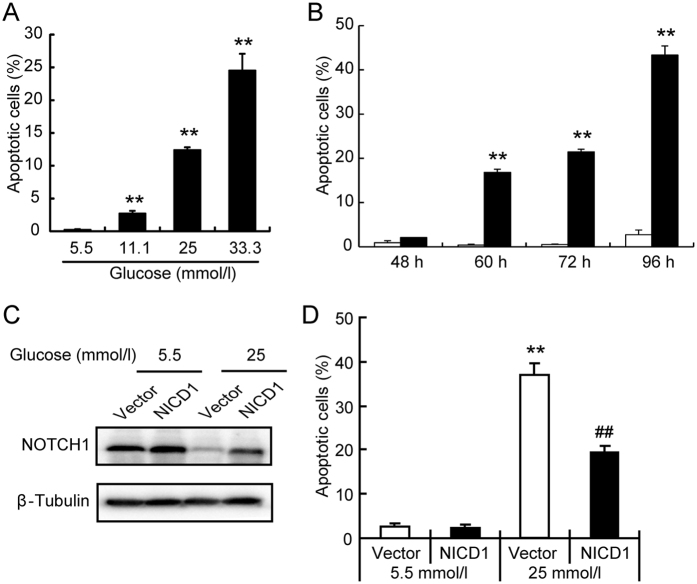
Inactivation of Notch1 caused beta cell apoptosis. INS-1 cells were treated with low glucose (5.5 mmol/l glucose, control), or high glucose (11.1, 25, 33.3 mmol/l glucose) for 60 h, followed by PI staining and flow cytometry analysis (**A**). INS-1 cells were treated with low glucose (5.5 mmol/l glucose, control, white bar), or high glucose (25 mmol/l glucose, black bar) for the indicated times, followed by PI staining and flow cytometry analysis (**B**). After transient transfection with vector (white bar) or an NICD (black bar) plasmid for 24 h, INS-1 cells were treated with glucose for 24 h when the protein levels of cleaved Notch1 were analyzed (**C**) and for 72 h the INS-1 cells were stained with propidium iodide (PI) and analyzed by flow cytometry (**D**). Data shown are means ± SEM and representative of three separate experiments. (**A,B**) **p < 0.01 vs. control. (**D**) **p < 0.01 vs. Vector +5.5 mmol/l glucose, and ^##^*p* < 0.01 vs. Vector +25 mmol/l glucose.

The glucotoxic condition has been associated with a gradually increased impairment of pancreatic beta cells, characterized as dysfunction in insulin secretion and defects in insulin biosynthesis in both rat primary islets and INS-1 cells (Supplementary Fig. 1). Moreover, prolong exposure of INS-1 cell to high concentrations of glucose enforced cellular apoptosis in a dose- (Fig. 2A) and time- (Fig. 2B) dependent manner.

NICD1 was also overexpressed to restore the cellular level of active Notch1 following its decrease by glucotoxicity (Fig. 2C). The overabundance of NICD1 resulted in a partial, but dramatic, inhibition of glucotoxicity-induced cellular apoptosis (Fig. 2D); however, the recovery of the glucotoxicity-induced insulin secretion defect was not statistically significant (Supplementary Fig. 2). All the data suggest that restoration of NICD1 levels can prevent glucotoxicity-induced apoptosis of beta cells without improvements in defective insulin secretion.

### MiR-375 targeted Psen1, miR-30a targeted Psen2, and miR-34a targeted Notch1

**Figure 3:**
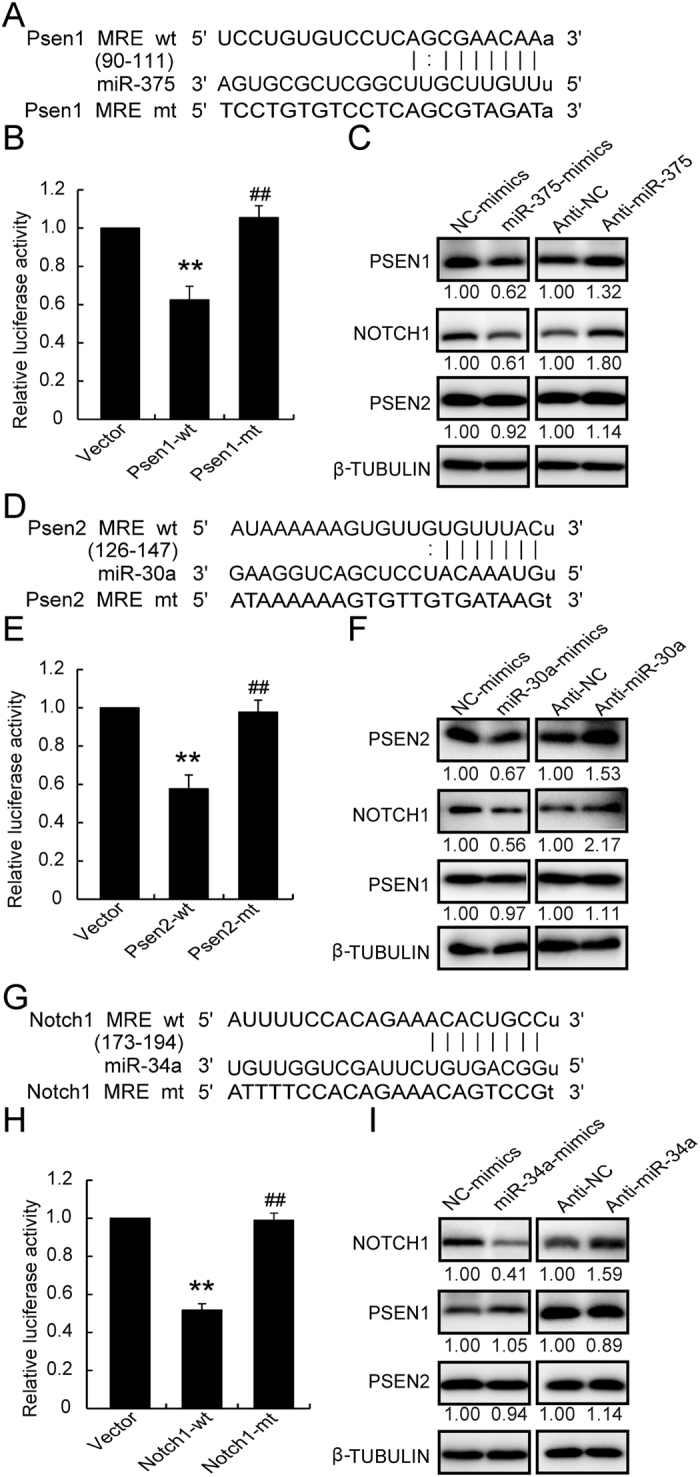
Target identification for miRNAs. The 3′utr sequence of *Psen1* (**A**), *Psen2* (**D**), and *Notch1* (**G**), predicted to include relative miRNA MREs, were aligned and both wt and mt sequences are listed. Wt and mt plasmids were cotransfected with relative miRNAs for 24 h, and luciferase reporter activities were analyzed for *miR-375* toward *Psen1* (**B**), *miR-30a* toward *Psen2* (**E**), and *miR-34a* toward *Notch1* (**H**). Protein levels were determined following overexpression or knockdown of *miR-375* (**C**), *miR-30a* (**F**), or by *miR-34a* (**I**). Data shown are means ± SEM and representative of three separate experiments. (**B**,**E**,**H**) **p < 0.01 *vs*. vector control, and ^##^*p* < 0.01 *vs*. relative wt plasmid.

The protein levels of *NOTCH1*, *PSEN1*, and *PSEN2* following cell culture with 11.1 mmol/l glucose were significantly decreased while that of mRNA levels were not altered suggesting the action of miRNAs. The combined use of bioinformatics prediction softwares (TargetScan and miRanda) and literature screening pointed to *miR-375*, *miR-30a*, and *miR-34a* as potential targets for further exploration. As shown in Fig. 3A,D,G, the miRNA response element (MRE) was well matched between *miR-375* and *Psen1*, between *miR-30a* and *Psen2*, and between *miR-34a* and *Notch1*. The luciferase activity assays showed that *miR-375* inhibited the activity of the wt-*Psen1* construct when compared with a vector control, whereas no alteration was observed with the mt-*Psen1* construct (Fig. 3B). Neither overexpression nor knockdown of *miR-375* altered the *Psen1* gene level (data not shown), whereas elevated *miR-375* repressed and downregulated *miR-375* enhanced PSEN1 protein level, consequently leading to the same protein expression profile for NICD1 (Fig. 3C). No alterations were observed with PSEN2 (Fig. 3C).

Luciferase activity assays, qRT-PCR, and western blotting were also performed to verify the relationships between *miR-30a* and *Psen2* and between *miR-34a* and *Notch1*. *MiR-30a* could target to the Psen2 3′utr region (Fig. 3E) and reduce the PSEN2 protein level (Fig. 3F) without impacting its mRNA level (data not shown). The reduction in PSEN2 also caused a reduction in NICD1 (Fig. 3F) without affecting PSEN1 level. Further, *miR-34a* could regulate the *Notch1* 3′utr region (Fig. 3H) and reduce the NOTCH1 protein level, without affecting PSEN1 and PSEN2 levels (Fig. 3I). No alteration of Notch1 mRNA level by *miR-34a* overexpression was observed (data not shown). Taken together, these results reveal that *miR-375* targets *Psen1*, *miR-30a* targets *Psen2*, and *miR-34a* targets *Notch1* by regulating the relevant 3′utr regions and repressing their translation, thereby suppressing the NICD1 level in INS-1 cells.

### *miR-375*, *miR-30a*, and *miR-34a* mimicked glucotoxicity-induced defects

**Figure 4:**
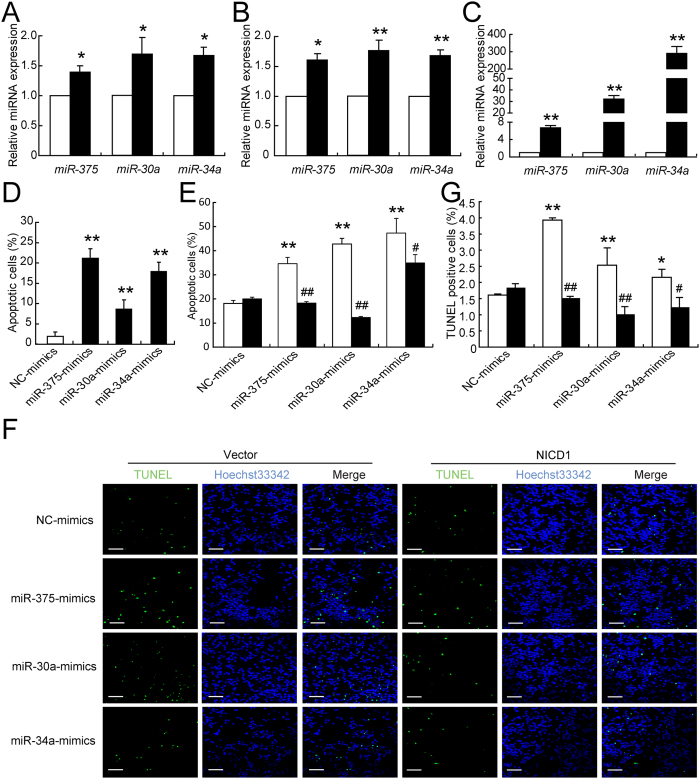
Effects of *miR-375*, *miR-30a*, and *miR-34a* expression of INS-1 cells. The expression levels of miRNAs were analyzed by qRT-PCR assays in islets treated with 11.1 (control) and 25 mmol/l glucose for 48 h (**A**), as well as in INS-1 cells treated with 5.5 (control) and 25 mmol/l glucose for 12 h (**B**). The expressions of *miR-375*, *miR-30a* and *miR-34a* were determined after 24 h miRNA transfection (**C**). Cellular apoptosis was analyzed 48 h after transfection (**D**). A vector (white bar) or NICD1 (black bar) plasmid was co-transfected with the indicated miRNAs or NC mimics for 48 h and the cells were then collected for PI staining and analyzed by flow cytometry (**E**). A vector or NICD1 plasmid was co-transfected with the indicated miRNAs or NC mimics for 48 h and TUNEL assay was carried out to detect dead cells. Scale bars = 40 μm (**F**). Quantitative result of TUNEL assay was analyzed. Data were obtained from 8 scans in each group (**G**). Data shown are means ± SEM and representative of three separate experiments. (**A**–**D**) *p < 0.05 or **p < 0.01 *vs*. control. (**E,G**) **p < 0.01 *vs*. NC + vector, and ^#^*p* < 0.05 or ^##^*p* < 0.01 *vs*. relative miRNA + vector.

The expression of *miR-375*, *miR-30a* and *miR-34a* was significantly induced by high glucose in both rat pancreatic islets (Fig. 4A) and INS-1 cells (Fig. 4B). The miRNA mimics were introduced to enforce cellular levels of *miR-375*, *miR-30a*, and *miR-34a* in INS-1 cells (Fig. 4C). Elevated levels of *miR-375*, *miR-30a*, and *miR-34a* decreased the K^+^-stimulated insulin secretion index (KSI) (SEM Fig. 3A), insulin content (SEM Fig. 3B), and insulin gene expression (SEM Fig. 3B), and increased the numbers of apoptotic cells (Fig. 4D). Interestingly, the overabundance of NICD1 resulted in a significant inhibition of miRNAs-induced cellular apoptosis (Fig. 4E–G); however, the recovery of these miRNAs-induced insulin secretion defect was not observed (data not shown). Taken together, these results suggest that *miR-375, miR-30a*, and *miR-34a* are capable of mimicking glucotoxicity-induced beta cell impairment and NOTCH1 inactivation associated with these miRNAs-induced beta cell apoptosis.

### Inhibiting *miR-375*, *miR-30a*, and *miR-34a* rescued glucotoxicity-induced defects

**Figure 5:**
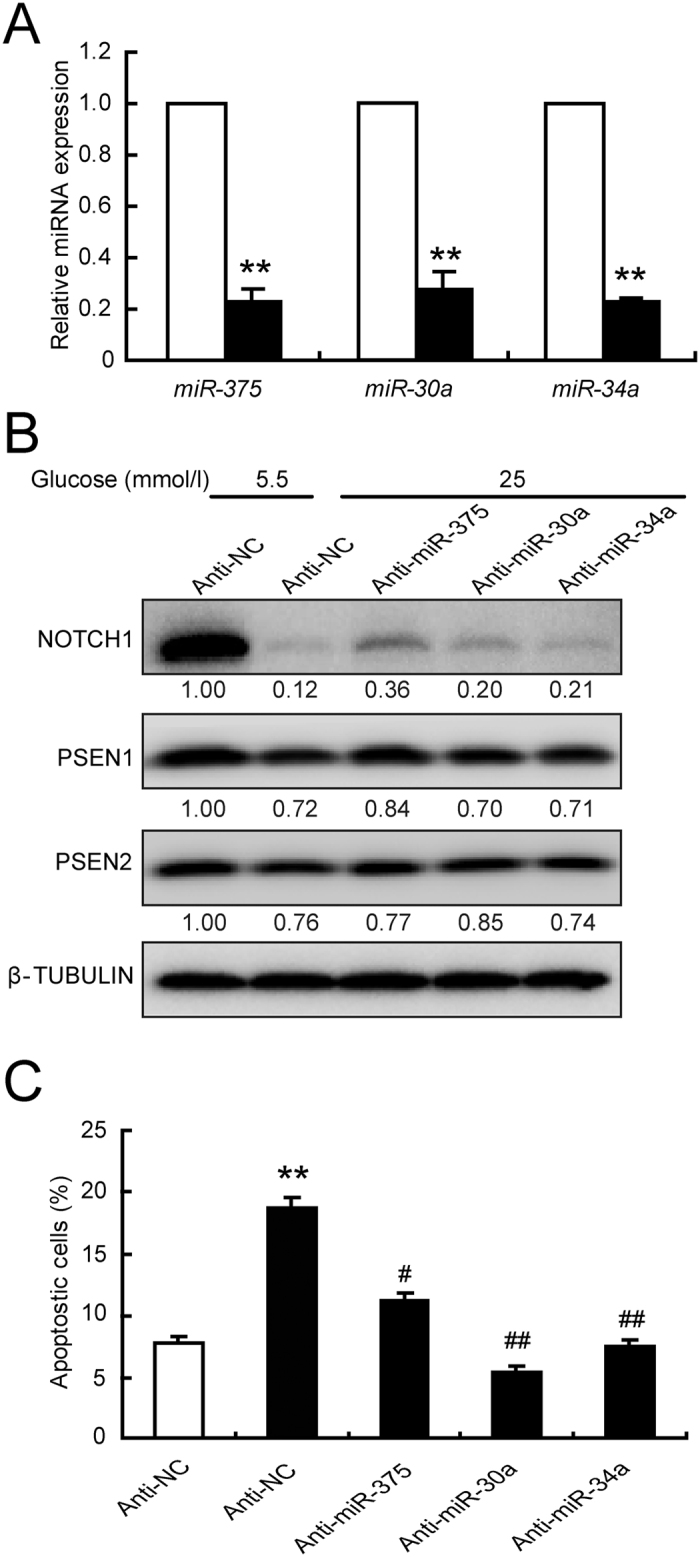
Inhibition of each of the three miRNAs recovered beta cell apoptosis. INS-1 cells were transfected with anti-NC (white bar, control) or the indicated anti-miRNAs (black bar) for 24 h, and the knockdown efficiency was determined by qRT-PCR (**A**). Transfected cells were allowed to equilibrate with 5.5 mmol/l glucose medium for 12 h, followed by an additional treatment with 5.5 (control) or 25 mmol/l glucose for the indicated times. After 24 h treatment, protein levels were determined (**B**). After 48 h treatment, apoptotic cells were analyzed by PI staining and flow cytometry (**C**). Data shown are means ± SEM and representative of three separate experiments. **p < 0.01 *vs*. control, and ^#^*p* < 0.05 or ^##^*p* < 0.01 *vs*. Anti-NC + 25 mmol/l glucose.

The function of these three miRNAs was further clarified by knocking down their expression levels in INS-1 cells using miRNA inhibitors (Fig. 5A). The expression level of *miR-375* was downregulated to 22.9%, that of *miR-30a* was downregulated to 28.1%, and that of *miR-34a* to 22.7% after anti-miRNAs transfection. The repression of PSEN1 by high glucose was recovered by anti-*miR-375*, repression of PSEN2 was recovered by *anti-miR-30a*, and repression of NOTCH1 was recovered by anti-*miR-34a*; all of these responses resulted in the recovery of NICD1 protein level (Fig. 5B). Moreover, all three anti-miRNAs effectively rescued the apoptosis induction (Fig. 5C) caused by high glucose. These data demonstrated that inhibition of *miR-375*, *miR-30a*, and *miR-34a* can partially rescue the beta cell apoptosis caused by glucotoxicity.

### Reciprocal regulation between miRNAs and Notch1 signaling in diabetic GK rats and aged Wistar rats

**Figure 6:**
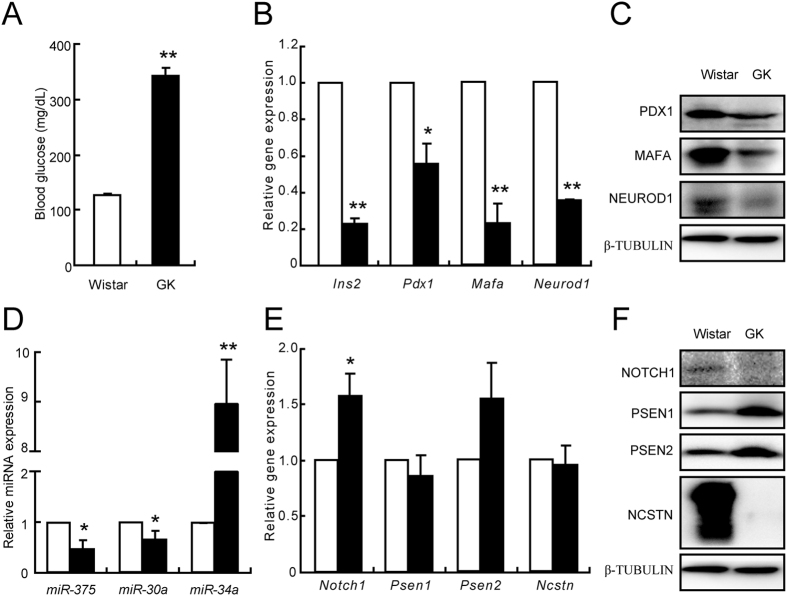
Alterations of genes and miRNAs in GK rats. Blood glucose levels in Wistar rats (white bar, control) and GK rats (black bar) (**A**). mRNA (**B**) and protein (**C**) levels of genes specific for beta cells. *miR-375*, *miR-30a* and *miR-34a* expression levels (**D**). mRNA (**E**) and protein (**F**) levels of NOTCH1 and components of γ-secretase. Data shown are means ± SEM. *p < 0.05 or **p < 0.01 *vs*. control. n = 6 per group.

The possibility that this regulation model also exists *in vivo* was evaluated using primary islets isolated from diabetic GK rats and aged Wistar rats. The random blood glucose levels were higher in GK rats than in age-matched control Wistar rats, confirming that the GK rats were diabetic (Fig. 6A). Analysis of the beta cell specific genes *Ins2*, *Pdx1*, *Mafa*, and *Neurod1* confirmed that the mRNA expression were significantly decreased in GK rats (Fig. 6B). The protein levels of PDX1, MAFA, and NEUROD1 were also dramatically reduced in the GK rats (Fig. 6C). The deficiencies in beta cell markers further confirmed a molecular basis for the diabetic phenotype of the GK rats. These GK rats also showed that *miR-375* and *miR-30a* expressions were decreased, while *miR-34a* expression was significantly increased (Fig. 6D). The mRNA level of *Notch1* was increased, while no alterations were observed in *Psen1*, *Psen2*, and *Ncstn* in the GK rats (Fig. 6E). Although the protein levels of PSEN1 and PSEN2 were significantly increased, that of NCSTN, another component of γ-secretase was dramatically decreased (Fig. 6F), suggesting a defective γ-secretase in islets isolated from GK rats. The decreased NOTCH1 protein level (Fig. 6F) further confirmed this defect in the γ-secretase in GK rat islets.

**Figure 7:**
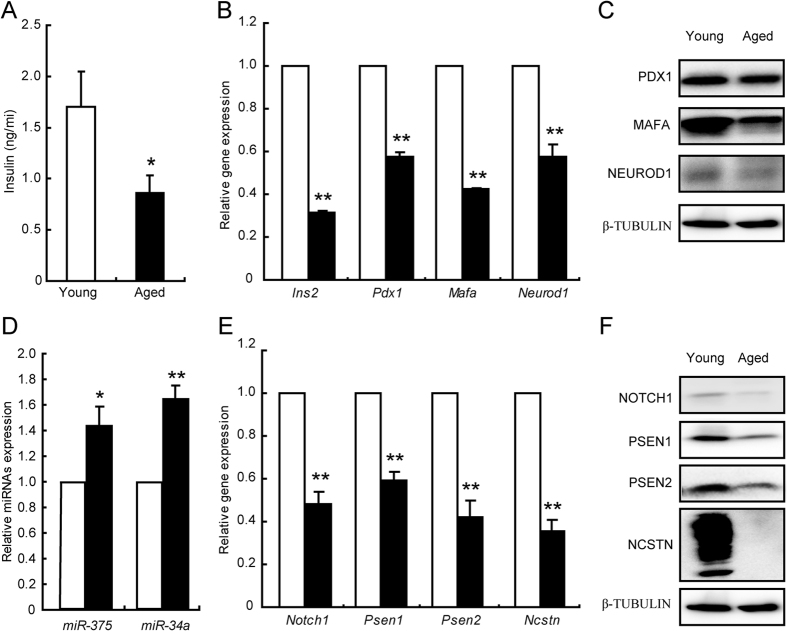
Alterations of genes and miRNAs in aged Wistar rats. Plasma insulin levels in young rats (white bar, control) and aged rats (black bar) (**A**). mRNA (**B**) and protein (**C**) levels of genes specific in beta cells were examined in islets isolated from young rats (white bar, control) and aged rats (black bar). *miR-375* and *miR-34a* expression levels (**D)**. mRNA (**E**) and protein (**F**) levels of NOTCH1 and components of γ-secretase. Data shown are means ± SEM. *p < 0.05 or **p < 0.01 *vs*. control. n = 6 per group.

The random plasma insulin levels were significantly lower in aged rats than in young Wistar rats, suggesting an impairment of insulin secretion in islets from aged rats (Fig. 7A). Aged rats also showed defective expressions of beta cell specific markers (Fig. 7B,C). The levels of *miR-375* and *miR-34a* were modestly elevated (Fig. 7D), in parallel with the decreased gene expression levels of components of γ-secretase and *Notch1* (Fig. 7E). The protein levels were also significantly decreased (Fig. 7F), indicating a defective γ-secretase in islets from aged rats. Taken together, the data demonstrated that alteration of miRNA expression disrupted the γ-secretase-mediated Notch1 cleavage which was associated with beta cell impairment.

## Discussion

This study was undertaken to elucidate the regulatory networks operating between miRNAs (*miR-375*, *miR-30a*, and *miR-34a*) and the presenilin/Notch1 pathway in the context of glucotoxicity-induced pancreatic beta cell impairment. Our research generated three major findings. First, we demonstrated that exposure to high glucose levels decreases the amount of NICD1, the active form of Notch1, by disruption of the γ-secretase-mediated Notch1 proteolytic cleavage pathway. Second, miRNAs function at the posttranscriptional level to play key roles in regulating Notch1 signaling. Specifically, *miR-375* targets *Psen1*, *miR-30a* targets *Psen2*, and *miR-34a* targets *Notch1* itself, but they all decrease the NICD1 level and inactivate Notch1 signaling. The third main observation is that a reciprocal modulation pattern occurs between miRNAs and the presenilin/Notch1 pathway in diabetic GK rats as well as in aged rats, suggesting that the *in vitro* regulatory network is likely to be functional *in vivo* and may contribute to the development of type 2 diabetes.

Glucotoxicity is an important factor that contributes to advancing beta cell failure and development of overt diabetes. We have demonstrated that glucotoxicity induces beta cell failure by inhibition of the γ-secretase-mediated Notch1 cleavage pathway. Early studies of Notch1 signaling focused on the regulation of pancreatic cell fate determination during development^24^, with later reports showing that Notch1 signaling regulates apoptosis in several mature cell types^25^,^26^,^27^. Interestingly, Darville *et al*. has pointed that the Notch1 signaling pathway induction by cytokines is likely to contribute to beta cell de-differentiation^15^. Dror *et al*. documented that the Notch cleavage pathway mediated by γ-secretase remained intact and functional in mature islets^14^. Suppression of Notch signaling, as indicated by decreased NICD1 levels, results in an increase in the Bax/Bcl-2 ratio, which consequently leads to cellular apoptosis through the caspase 3-dependent pathway^28^. An increase in the Bax/Bcl-2 ratio has a well-known association with glucotoxicity-induced apoptosis in several cell types, including beta cells^29^. Therefore, a decreased level of NICD1 will potentially act as a trigger of glucotoxicity-induced impairment in beta cells.

NICD1, the active form of Notch1, is generated by γ-secretase-mediated proteolytic cleavage^10^. Therefore, blocking either this cleavage pathway or Notch1 itself results in decreased NICD1 levels. Indeed, the components of the γ-secretase proteolytic complex were significantly decreased by high glucose in our study. The effects of presenilins have been comprehensively studied in genetic Alzheimer disease because of their contribution to the processing of the amyloid precursor protein (APP)^30^. Diabetes and Alzheimer disease share risk factors, and it is possible that these two diseases may share common molecular defects.

Although presenilins are key mediators in neurodegeneration, their role in beta cell degeneration remains unclear. Previous studies have shown that presenilin levels can be increased by low concentration of glucose (2 mmol/l glucose for PSEN1, and 5 mmol/l glucose for PSEN2), resulting in defective intercellular Ca^2+^ homeostasis and beta cell death due to low metabolic activity^12^. Consistent with these findings, the protein levels of presenilins are also most abundant at low glucose levels. However, our results suggest that the decreased levels of presenilins due to high glucose lead to reductive cleavage of Notch1 and ultimately to beta cell apoptosis.

The protein levels of presenilins and NOTCH1 are significantly decreased by high glucose, and yet their mRNA levels are only slightly altered, supporting a role for miRNAs as the key regulators underlying the deficiency in the Notch1 signaling pathway. Previously, upregulation of *miR-375* and *miR-30a* expression by glucotoxicity has been reported in rat islets and INS-1 cells^17^. Elevated serum levels of *miR-375* and *miR-30a* are also observed in diabetic patients^31^,^32^. In the present study, *miR-375* expression increased initially but was significantly decreased by high glucose over time. The alteration in this expression pattern for *miR-375* is related to the transcriptional activity of PDX1, since *miR-375* is one of its target genes^33^. Several reports have demonstrated that *miR-34a* expression is increased in primary islets from high-fat-diet (HFD) mice and genetic diabetic *db/db* mice, as well as in beta cells treated with cytokines and palmitate^16^,^23^. Elevated levels of *miR-34a* were also observed in the present study in INS-1 cells stimulated by high glucose and in primary islets from diabetic GK rats and aged rats. The regulatory network between *miR-34a* and *p53* has been confirmed by several groups^34^,^35^,^36^. The transcriptional activity of p53 is promoted by high glucose in INS-1 cells (Our unpublished data). Therefore, the elevated levels of *miR-34a* observed are likely an effect of triggering of p53 activity by high glucose in our study, as *miR-34a* is a conventional target gene of p53.

The present study and other studies have shown that elevation of *miR-375*, *miR-30a*, or *miR-34a* individually results in beta cell dysfunction, decreases cell survival, and increases apoptosis, thereby mimicking the cellular phenotype provoked by glucotoxicity. A combination of literature screening, bioinformatics analysis, and experimental assays confirms that *miR-375*, *miR-30a*, and *miR-34a* regulate NICD1 protein expression via diverse targets involving γ-secretase-mediated Notch1 cleavage. However, Notch1 signaling is only associated with glucotoxicity-induced beta cell apoptosis and its effects are unlike those evoked by *miR-375*, *miR-30a*, and *miR-34a*.

These differences are likely to result from the differences in the target proteins they modulate. Indeed, previous research has elucidated that *miR-375* regulates the expression of MTPN and PDK1 proteins^19^,^20^, while Kim *et al*. have claimed that *miR-30a* silencing of NEUROD1 expression is an important initial event of glucotoxicity-induced beta cell dysfunction^17^. *MiR-34a* targets VAMP2 and BCL2 expression, leading to beta cell damage^23^,^37^. In the present study, we showed that *miR-375* targets *Psen1*, *miR-30a* targets *Psen2*, and *miR-34a* targets *Notch1*, all of which are newly identified targets that converge onto one signaling pathway. To our knowledge, this effective modulation network has never been reported before.

Previous studies have elucidated the role of single miRNAs regulating different targets. For instance, we have reported that *miR-24* synchronously regulates four MODY (Mature Onset of Diabetes of the Young) genes^16^. Other reports have shown the regulation of one target by two or more miRNAs. Guttilla *et al*. have shown that FOXO1 can be coordinately regulated by *miR-27a*, *miR-96*, and *miR-182*^38^. Nevertheless, our study is the first to demonstrate that multiple miRNAs hierarchically regulate one pathway through diverse targets. This regulatory network would somehow insure that the cells receive the correct signal for committing to a cellular response. The use of INS-1 cells might raise a cautionary concern in interpreting our results, but a reciprocal correlation was noted *in vivo* between miRNAs and NICD1 in both diabetic GK rats and aged rats.

In conclusion, we have shown that *miR-375*, *miR-30a*, and *miR-34a* orchestrate the regulation of γ-secretase-mediated Notch1 cleavage in a glucotoxicity setting in pancreatic beta cells. This miRNA/Notch1 signaling pathway is likely to be related to beta cell impairment in mature pancreatic islets in rodents. This study provides a more comprehensive view of miRNAs, broadens our understanding of the pathophysiological development of diabetes, and identifies a fine-tuned regulatory network of miRNAs in disease.

## Materials and Methods

### Isolation of pancreatic islets

All animal studies performed were approved by the Research Animal Care Committee of Nanjing Medical University. Animals were treated humanely, using approved procedures in accordance with the guidelines of the Institutional Animal Care and Use Committee at Nanjing Medical University, China. Male Sprague-Dawley (SD) rats were purchased from Shanghai Laboratory Animal Centre (Chinese Academy of Sciences, China). Male Goto-Kakizaki (GK) and Wistar rats were purchased from Cavens Laboratory Animal Co., Ltd. (Chang Zhou, China). Islet isolation and culturing techniques have been described previously^16^.

### Cell culture

The rat insulinoma INS-1 cell line was cultured in RPMI-1640 (Invitrogen, Carlsbad, CA) supplemented with 10% FBS, 1% penicillin-streptomycin, 1 mM pyruvic acid sodium, 10 mM HEPES, and 0.05 mM β-mercaptoethanol (Sigma-Aldrich, St. Louis, MO) and incubated in a Thermo tissue-culture incubator at 37° with a 95% O_2_/5% CO_2_ atmosphere. Cells between passages 16 and 32 were used for experiments^39^.

### Plasmid construction

The primers for generating wide-type (wt) and mutant (mt) 3′utr-luciferase constructs of *Psen1*, *Psen2*, and *Notch1* are given in Supplementary information. To generate the wide-type (wt) and mutant (mt) 3′UTR-luciferase constructs of *Psen11*, *Psen2* and *Notch1*, the primers in Supplementary Table 1 were designed and synthesized by Generay Biotech Co., Ltd (Shanghai, China). Primers were annealed by incubating at 95° for 5 min, then heated at 70° for 10 min and allowed to cool down to room temperature. The generated double-stranded oligonucleotides were treated with T4 polynucleotide kinase (Promega) by incubating at 37° for 30 min, then heated at 70° for 10 min and transfered on ice. Wt or mt sequences were subcloned into the SpeI and HindIII sites of the pMIR-REPORT vector.

### Transient transfection and luciferase reporter assay

INS1 cells (1 × 10^4^ cells/well) were cultured in 48-well plates and co-transfected with luciferase reporter plasmids (either a WT or mutant plasmid) and miRNAs or the NC (negative control) mimics (GenePharma, Shanghai, China) using Lipofecatamine 2000 (Invitrogen, Carlsbad, CA) according to the manufacturer’s instructions. Twenty-four hours after transient transfection, the cells were harvested for dual luciferase reporter assays as described previously. The Firefly luciferase activity was normalized with the Renilla activity of the PRL-CMV plasmid (Promega, Madison, WI).

### Insulin secretion assay

Insulin secretion was determined in INS-1 cells and islets from SD rats. INS-1 cells (1 × 10^4^ cells/well) and isolated islet from SD rats (8 islets/well) in 48-well plates were cultured in the presence of different concentration of glucose (5.5, 11.1, 25 and 33.3 mmol/l) for 24 h in INS-1 cells and for 72 h in islets. INS1 cells were used for potassium-stimulated insulin secretion (KSIS) assays, islets were used for GSIS assays. The insulin levels were measured using a [^125^I] Insulin Radioimmunoassay Kit (North Institute of Biological Technology, Beijing, China)^40^.

### Flow cytometry analysis

INS1 cells (1 × 10^6^ cells/well) were cultured in 35 mm dishes and treated as described previously. The cells were harvested and fixed with 1 mL 75% ice-cold ethanol at −20° overnight. Cells were stained with propidium iodide (PI) solution (50 mg/ml) containing RNase (25 mg/ml) for 30 min in the dark at 37° and analyzed by using a FACSCalibur Flow Cytometer (Becton Dickinson, San Jose, CA).

### TUNEL assay

A TUNEL assay was performed with the commercially available *in situ* cell apoptosis detection kit (Vazyme, Nanjing, china), according to the manufacturer’s instructions. The TUNEL signals were observed with laser scanning confocal microscope (FV1200, Olympus, Japan). In each sample, eight scans were selected and the cells positive with TUNEL staining (green signals) and hoechst33342 staining (blue signals) were counted. Quantitative assessment of apoptosis was determined as a ratio of the number of TUNEL-positive cells to total nuclear number in each field.

### qRT-PCR analysis

Total RNA was extracted using Trizol reagent (Invitrogen). For miRNA quantitation, stem-loop primers designed to have a short single-stranded part that is complementary to the 3′-end of miRNA, a double-stranded part (the stem) and the loop that contains the universal primer-binding sequence were used as reverse primers. For mRNA determination, oligo-dT was used as reverse primer. Then, first-strand cDNA synthesis was performed using 1 μg of total RNA (Promega). All Reverse transcriptase reactions included no-template controls and RT minus controls. Quantitative RT-PCR was performed using the SYBR Green PCR Master Mix and LighteCycler480 Sequence Detection System (Roche). MiRNAs were normalized with u6 and mRNA was normalized with β-actin. Primers for quantitative RT-PCR were available in Supplemental Table 2.

### Western blot analysis

Cells or primary islets were lysed with ice-cold lysis buffer containing: 50 mmol/l Tris-HCl, pH 7.4, 1% NP-40, 150 mmol/l NaCl, 1 mmol/l EDTA, 1 mmol/l phenylmethylsulphonyl fluoride, and complete proteinase inhibitor (one tablet/10 ml; Roche). After protein content determination, western blot was performed as described before. Individual immunoblots were probed with antibodies to NOTCH1, NCSTN, PEN2, PSEN1 and PSEN2 (Cell Signalling, Danvers, MA) diluted 1:1000; to PDX1 (Upstate) diluted 1:3000; to MAFA (Santa cruz,) diluted 1:800; to NEUROD1 (abcam) diluted 1:3000. β-tubulin antibody (Sigma-Aldrich, St. Louis, MO) diluted 1:5000 was used as a standard.

### Statistical analysis

Comparisons were performed using the Student’s *t* test between two groups or ANOVA in multiple groups. The results are presented as means ± SEM. A *p* value of less than 0.05 was considered to be statistically significant.

## Additional Information

**How to cite this article**: Li, Y. *et al*. A Presenilin/Notch1 pathway regulated by *miR-375*, *miR-30a*, and *miR-34a* mediates glucotoxicity induced-pancreatic beta cell apoptosis. *Sci. Rep*. **6**, 36136; doi: 10.1038/srep36136 (2016).

**Publisher’s note:** Springer Nature remains neutral with regard to jurisdictional claims in published maps and institutional affiliations.

## Supplementary Material

Supplementary Information
