# Supplementary material for: A Presenilin/Notch1 pathway regulated by miR-375, miR-30a, and miR-34a mediates glucotoxicity induced-pancreatic beta cell apoptosis
Source: Sci Rep. 2016 Nov 2;6:36136. doi: 10.1038/srep36136 (PMC5095347; doi:10.1038/srep36136)
Supplement: Supplementary Information [file srep36136-s1.pdf]

SUPPLEMENTARY INFORMATION

A Presenilin/Notch1 pathway regulated by *miR-375*, *mR-30a*, and *miR-34a* mediates glucotoxicity induced-pancreatic beta cell apoptosis

Yating Li, Tao Zhang, Yuncai Zhou, Yi Sun, Yue Cao, Xiaoai Chang, Yunxia Zhu, and Xiao Han

Supplementary Table 1 Primers for wt and mt 3'utr plasmids cloning.

NAME	SEQUENCES (5'-3')	
	Forward	Reverse
wt-*Psen1*	CTAGT TCCTGTGTCCTCAGCGAACAAA A	AGCTT TTTGTTCGCTGAGGACACAGGA A
mt-*Psen1*	CTAGT TCCTGTGTCCTCAGCGTAGATA A	AGCTT TATCTACGCTGAGGACACAGGA A
wt-*Psen2*	CTAGT ATAAAAAAGTGTTGTGTTTACT A	AGCTT AGTAAACACAACACTTTTTTAT A
mt-*Psen2*	CTAGT ATAAAAAAGTGTTGTGATAAGT A	AGCTT ACTTATCACAACACTTTTTTAT A
wt-*Notch1*	CTAGT TTTTCCACAGAAACACTGCCT A	AGCTT AGGCAGTGTTTCTGTGGAAA A
mt-*Notch1*	CTAGT TTTTCCACAGAAACAGTCCGT A	AGCTT ACGGACTGTTTCTGTGGAAAA A

Supplementary Table 2 Primers for quantitative RT-PCR.

NAME	SEQUENCES (5'-3')	
	**Forward**	**Reverse**
Notch1	GGTGCGAGCGCAGTGAAGGA	CCCGCTGCTGCCCTCTTTCC
Psen1	CAAGAGCTGCTGTCCAGGAA	TGAAAATGGCGAGCAGGAGT
Psen2	TGTACGATCTCGTGGCTGTG	TCCATCTCTGGATCGTAAGGGA
Ncstn	CTGGGCCACAGAGACGATG	CTCCACTGAGTTTCCCCCAC
Psenen	GAAGGGGTAAACCCCACTG	GTACTTCCGGCACAGGTTCA
Ins1	CATAGACCATCAGCAAGCAGG	GAAGAAACCACGTTCCCCAC
Ins2	TGTCAAACAGCACCTTTGTGG	GTGCCAAGGTCTGAAGGTCAC
Pdx1	GGTATAGCCAGCGAGATGCT	TCAGGTGGGAGCCTGATTCT
Mafa	AAGGAGGAGGTCATCCGACT	TCTGGAGCTGGCACTTCTCG
Neurod1	ATGACCAAATCATACAGCGAGAG	TCTGCCTCGTGTTCCTCGT
Actb	GCCATGTACGTAGCCATC	CTCTCAGCTGTGGTGGTG
miR-375	ACACTCCAGCTGGGTTTGTTCGTTCGGCTC	CTCAACTGGTGTCGTGGAGTCGGCAATTCAGTTGAGTCACGCGA
miR-34a	ACACTCCAGCTGGGTGGCAGTGTCTTAGCTG	CTCAACTGGTGTCGTGGAGTCGGCAATTCAGTTGAGAACAACCA
Urp		TGGTGTCGTGGAGTCG
u6	CTCGCTTCGGCAGCACA	AACGCTTCACGAATTTGCGT

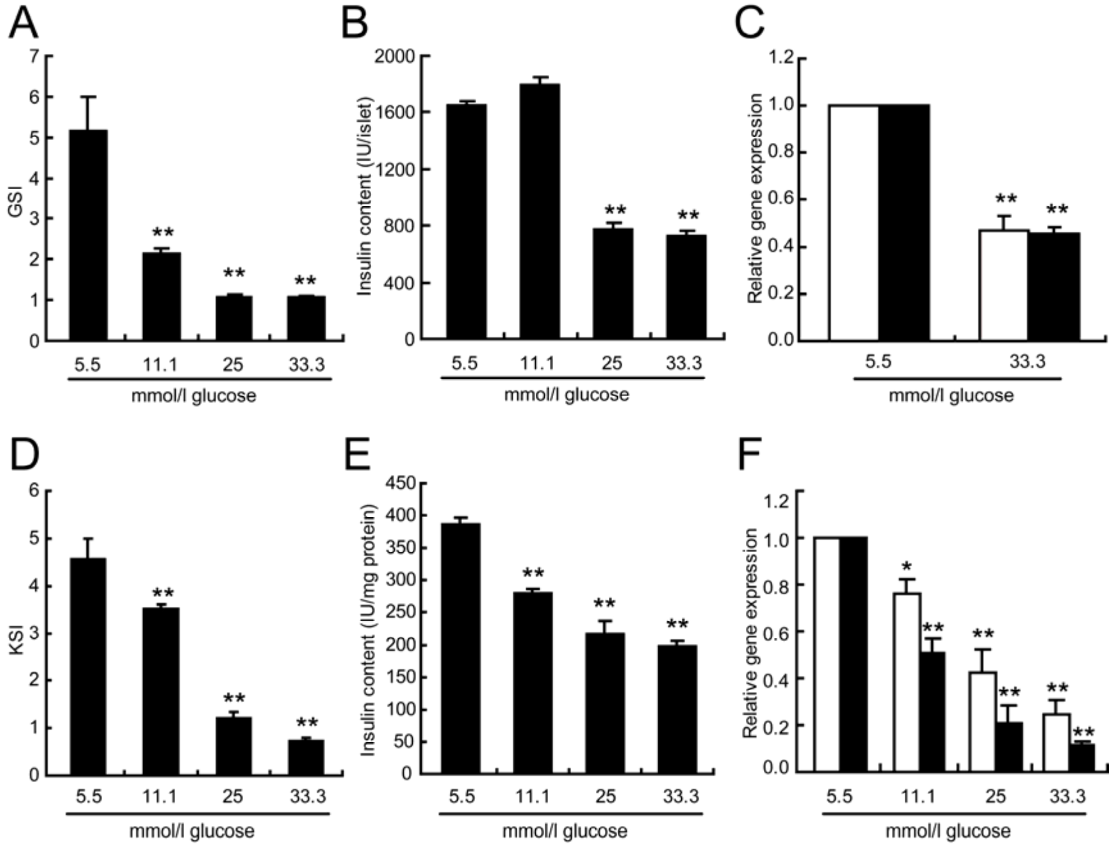

Supplementary Figure. 1 Glucotoxicity caused beta-cell dysfunction. (**A-C**) Rat islets were treated with low glucose (5.5 mmol/l glucose, control), or high glucose (11.1, 25, 33.3 mmol/l glucose) for 72 h by which time glucose-stimulated insulin secretion assay as shown by GSI (**A**), insulin content (**B**), as well as *Ins1* (white bars) and *Ins2* (black bars) genes level (**C**) analyzed by qRT-PCR were accessed. (**D-F**) INS-1 cells were treated with low glucose (5.5 mmol/l glucose, control, white bars), or high glucose (11.1, 25, 33.3 mmol/l glucose, blark bars) for 24 h by which time potassium-stimulated insulin secretion assay as shown by KSI (**A**), insulin content (**B**), as well as *Ins1* (white bars) and *Ins2* (black bars) (**C**) analyzed by qRT-PCR were accessed. Data shown are means ± SEM and representative of three separate experiments. *p<0.05 and **p<0.01 *vs.* control.

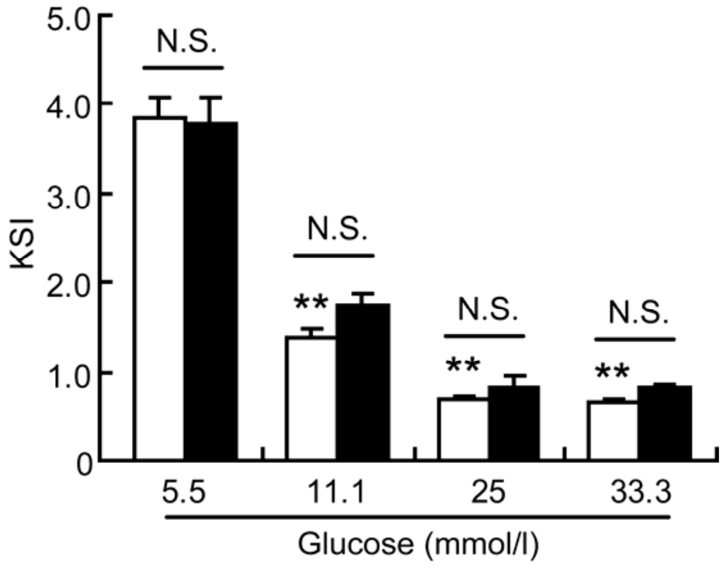

Supplementary Figure 2. Overexpression of NICD1 could not recover glucotoxicity-induced insulin secretion defect. After transient transfection with vector (control, white bar) or NICD (black bar) plasmid for 24 h, INS-1 cells were further treated with glucose for 24 h and KSIS was performed. Data shown are means ± SEM and representative of three separate experiments. ***p*<0.01 *vs.* control.

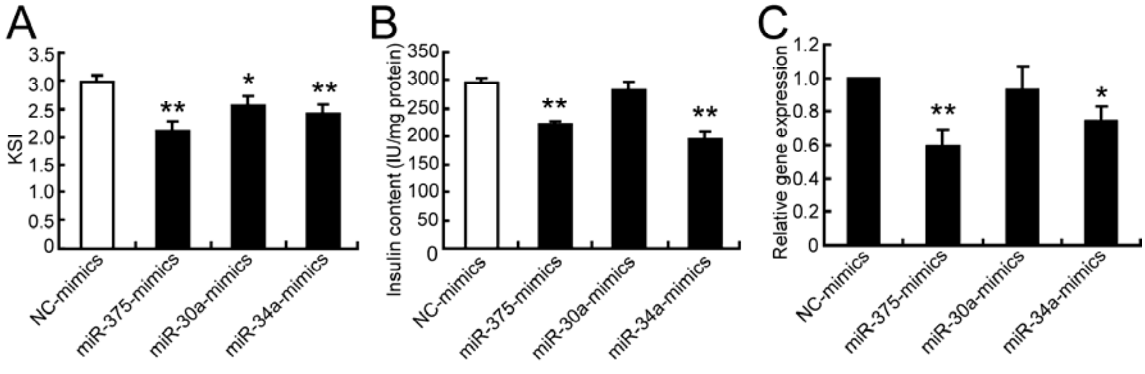

Supplementary Figure 3. *miR-375, miR-30a* and *miR-34a* induced beta cell dsyfunction in INS-1 cells. INS-1 cells were transfected with *miR-375*, *miR-30a* and *miR-34a* for 24 h when KSI (**A**), insulin content (**B**) and *Ins2* gene level (**C**) were determined. Data shown are means ± SEM and representative of three separate experiments. *p<0.05 and **p<0.01 *vs.* control.

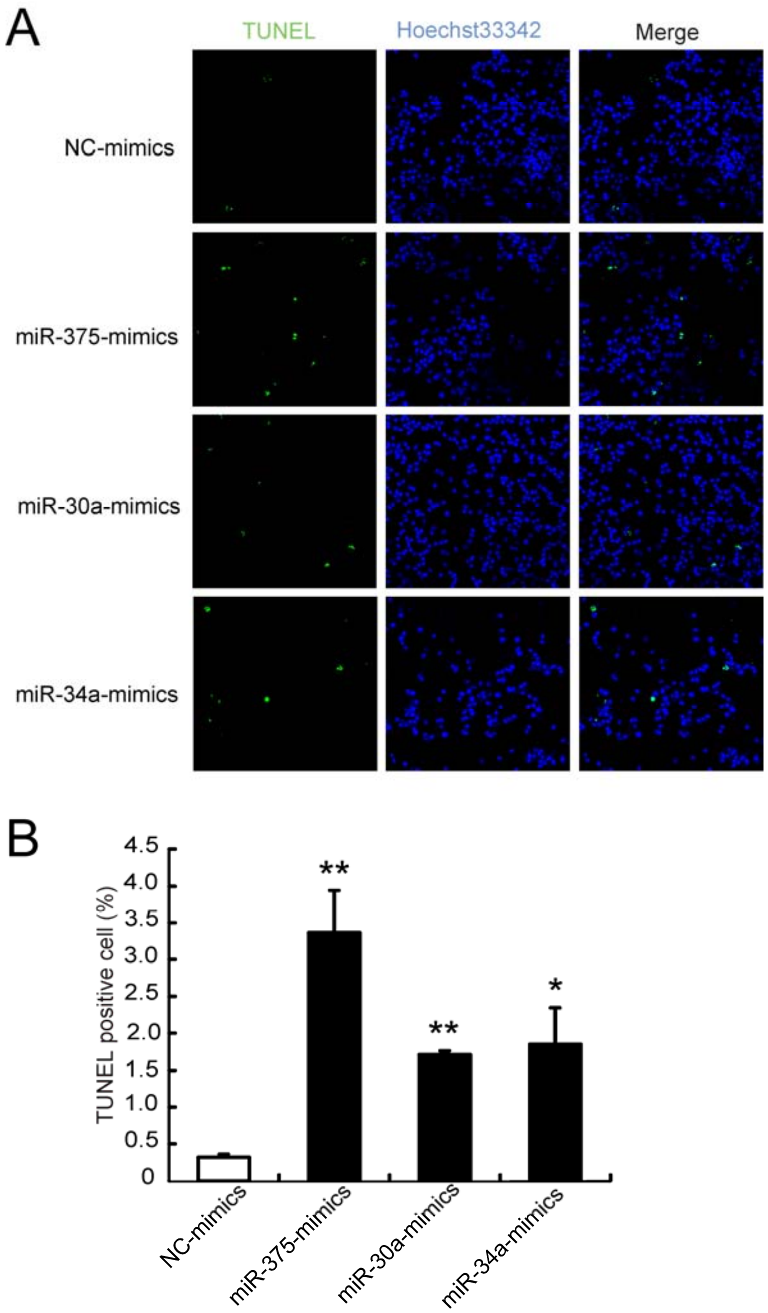

Supplementary Figure 4. *miR-375*, *miR-30a* and *miR-34a* induced beta cell apoptosis in INS-1 cells. INS-1 cells were transfected with *miR-375*, *miR-30a* and *miR-34a* for 48 h whenTUNEL assay was performed (**A**), and quantitative result of TUNEL assay was analyzed (**B**). Data were obtained from 8 scans in each group *p<0.05 and **p<0.01 *vs.* control.

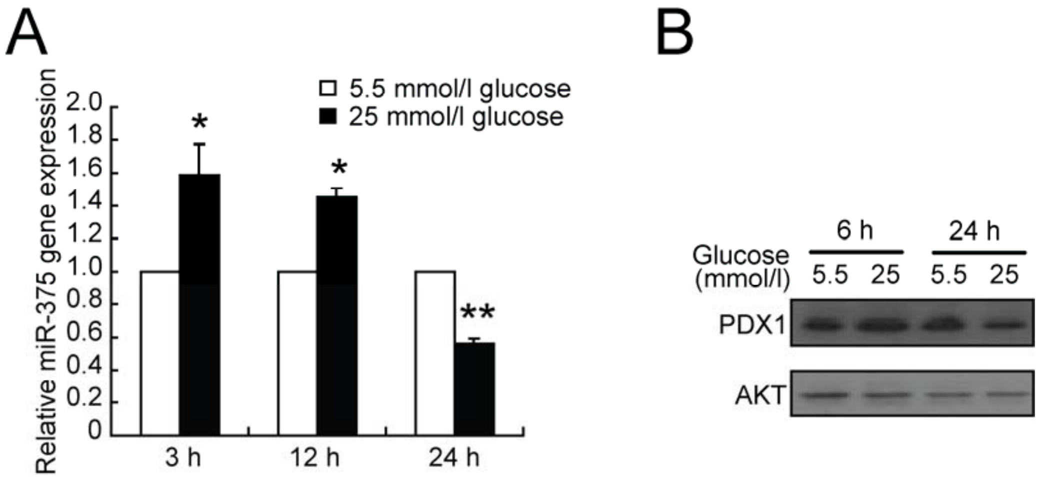

Supplementary Figure. 5 The inverted regulation between PDX1 and miR-375 by glucotoxicity. INS-1 cells were treated with 5.5 or 25 mmol/l glucose for indicated time when expression level of miR-375 was detected by qRT-PCR (A). INS-1 cell were exposure to 5.5 or 25 mmol/l glucose for indicated time when protein level of PDX1 was measured (B). AKT was used as an internal standard. Data shown are means ± SEM and representative of three separate experiments. *p<0.05 and **p<0.01 *vs.* control.
